# Comparison and characterization of the bacterial microbiota and SIgA production in different gastrointestinal segments in horses

**DOI:** 10.1007/s11259-024-10489-8

**Published:** 2024-08-24

**Authors:** Agnieszka Żak-Bochenek, P. Żebrowska-Różańska, J. Bajzert, N. Siwińska, J. P. Madej, K. Kaleta-Kuratewicz, P. Bochen, Ł. Łaczmański, A. Chełmońska-Soyta

**Affiliations:** 1 Department of Immunology, Pathophysiology and Veterinary Preventive Medicine, Faculty of Veterinary Medicine, Wrocław University of Environmental and Life Sciences, C. Norwida 31, 50-375 Wrocław, Poland; 2 Laboratory of Genomics and Bioinformatics, Hirszfeld Institute of Immunology and Experimental Therapy, Polish Academy of Sciences, Weigla 12, 53-114 Wrocław, Poland; 3 Department of Internal Diseases and Clinic of Diseases of Horses, Dogs and Cats, Faculty of Veterinary Medicine, Wrocław University of Environmental and Life Sciences, C. Norwida 31, 50-375 Wrocław, Poland; 4 Department of Biostructure and Animal Physiology, Division of Histology and Embryology, Faculty of Veterinary Medicine, Wrocław University of Environmental and Life Sciences, C. Norwida 25, 50-375 Wrocław, Poland; 5 Laboratory of Medical Microbiology, Hirszfeld Institute of Immunology and Experimental Therapy, Polish Academy of Sciences, Weigla 12, 53-114 Wrocław, Poland

**Keywords:** Horses, Microbiome, Intestine, Secretory immunoglobulin A, Plasma cells

## Abstract

In the gastrointestinal mucosa, there is a close cooperation between secretory immunoglobulin A (SIgA) and the composition of the microbiota, which aims to maintain homeostasis as well as act as a protective barrier. The purpose of this study was to determine the composition of microbiota and SIgA production in different parts of the digestive tract (small intestine, cecum, colon and rectum) of nine healthy horses and its reflection in the feces. For this purpose, we determined: the composition of the microbiome (by next-generation Sequencing of Hypervariable Regions V3-V4 and V7-V9 of the *16 S rRNA* gene analysis), the amount of SIgA in the intestinal content samples (by ELISA), as well as the number of IgA-producing cells (IgA+) in the tissue samples (by immohistochemical analysis). Significant differences were observed between the small intestine and the large colon in the composition and diversity of the microbiome, as well as the number of IgA + cells in the mucosal lamina propria and the abundance of SIgA in the intestinal lumen. The small intestine in relation to the large colon is characterised by fewer IgA + cells, more SIgA in the intestinal contents and a less diverse microbiome. However, the cecum appears to be the third separate ecosystem, with a high number of IgA + cells and a diverse microbiome. The fecal sample reflects the current state of the large colon, both in terms of the microbiome and SIgA content; however, it is not known to what extent it may be influenced by dysbiosis in other parts of the digestive tract.

## Introduction

The gastrointestinal tract of mammals is the largest barrier to pathogens in the body as a mucosal firewall and generates an immune response within the gastrointestinal associated lymphoid tissue (GALT) (Chase and Kaushik 2019). The first line of defense is continuity of the mucosa within the small and large intestines. The mucosa consists of one layer of cylindrical cells, whose tight junctions act as physical barriers to microorganisms (Maynard et al. 2012). The cell surface is covered with two layers of mucus, inner and outer, which can be considered a defense against epithelial cells. The inner mucus layer is recognized in the literature as a “killing zone” and includes antimicrobial peptides suspended in the mucus and immunoglobulin A in the secretory form (SIgA) (Chase and Kaushik 2019). The outer mucosal layer is colonized by commensal bacteria known as the microbiome. The mucus layer prevents direct contact between bacteria (including commensal bacteria) and the surface of intestinal epithelial cells (IEC). However, there are reports in the literature of segmented filamentous bacteria (SFB) that, owing to their structure, can break through this barrier and attach to the IEC surface. Bacteria of the microbiome can interact with the host immune system. SFB has recently attracted attention because of its role in promoting adaptive and innate immunity in mice and rats through the differentiation and maturation of Th17 cells in the intestinal tract and SIgA production (Hedblom et al. 2018). However, other bacteria, such as the *Proteobacteria* phylum, also show strong cooperation with the host immune system and are responsible for the regulation of SIgA production in mice (Mirpuri et al. 2014).

The composition of the equine gastrointestinal microbiome has been described in many recent investigations, taking into account its division into anatomical regions and health and disease conditions (Costa et al. 2015; Ericsson et al. 2016; Su et al. 2020; Reed et al. 2021). However, the amount of SIgA in the gastrointestinal tract and its ability to produce SIgA by analyzing the number of IgA-producing (IgA+) plasmocytes in the lamina propria have not yet been studied in horses. Production of IgA in a dimeric form occurs in plasmocytes that inhabit the lamina propria of the mucosa. The transport of IgA dimers into the intestinal lumen is facilitated by binding to polymeric immunoglobulin receptors (pIgR) that are located at the basolateral membrane of intestinal epithelial cells and transported to the apical surface by transcytosis where the external domains of pIgR are cleaved from the intramembranous domains thereby releasing IgA bound to the so-called secretory fragment (S). In addition to pathogen neutralisation, SIgA regulates the composition of the gut microbiota and plays a role in maintaining the homeostatic relationship between the intestinal microbiota and the host (Pietrzak et al. 2020). Reduced intestinal SIgA production has been found in dogs suffering from inflammatory bowel disease (IBD), but little is known on this topic in horses (Maeda et al. 2013). To date, differences in IgA production in different parts of the gastrointestinal tract have also not been evaluated.

Reciprocal relationships exist between factors that maintain homeostasis and gastrointestinal health. The hypothesis stated at the beginning of the investigation was that the environment of the small intestine and large intestine in horses differs significantly, including in the manner of inter-kingdom interaction: the host vs. its microbiome and immune reaction. Understanding the physiology of both sections of the gastrointestinal tract is crucial for establishing preventive and therapeutic plans for horses with different intestinal inflammatory states. The second purpose of this study was to assess the extent to which a stool sample can reflect the condition of the gastrointestinal tract as a noninvasive method. To determine the relevance of the hypothesis, we analyzed the microbiome composition of intestinal contents and tissue samples, the level of SIgA in the content, and the number of IgA + cells in the mucosal lamina propria of five parts of the equine gastrointestinal tract: duodenum, ileum, ceacum, colon, and rectum in healthy horses.

## Materials and methods

### Study design

The experimental research plan included the collection of gastrointestinal (duodenum, ileum, cecum, large colon, and rectum) and fecal samples from a group of 9 healthy slaughtered horses. In luminal content samples, the composition of the microbiome and the amount of SIgA were examined. In mucosal samples, histopathological examination was performed, the number of IgA + cells was determined, and the composition of the microbiome was examined.

### Material

In this study gigestive tract samples collected from nine horses slaughtered in a slaughterhouse were used. The horses enrolled in the study were clinically healthy, under optimal conditions, of both sexes (7 mares, 2 stallions), and ranged from 2 to 14 years of age (mean 9.9, SD = 3.9). Before arriving at the slaughterhouse, the horses stayed at a breeding center, with a similar diet dedicated to draught horses. Horses were fed according to their energy needs, with concentrate (grain) and forage (hay) and had unlimited access to water and mineral licks. None of the horses received probiotic or prebiotic supplements or antibiotics for three months prior to slaughter. Transport to the slaughterhouse covered a distance of up to 100 km. Until slaughter, all horses spent 24–48 h in a stable for acclimatization, with access to hay and water. The slaughter was carried out in accordance with the rules and principles of animal welfare. Immediately after slaughtering the animals, the digestive tract was removed from each of the test animals, omitting the esophagus and stomach. Each fragment was examined macroscopically and then excised. Luminal content samples (LCS) and mucosal samples (MS) were taken from each of the fragments of interest, that is, duodenum (DUO), 30 cm from the pylorus; ileum (IL), 30 cm in front of the ileocecocolic cavity; cecum (CEC) - apex; large colon (COL) - pelvic flexion region; and rectum (REC). The luminal contents were scraped from the mucosa with a sterile spatula, placed in two 1.5 ml tubes, and immediately frozen at -80 °C. For the REC sample, the luminal content was also a fecal sample, secured as aboved. Mucosal samples (for microbiome analysis) after washing with sterile 0.9% NaCl solution were preserved in 500 µL RNA protect tissue reagent (Cat. No. 169014460) and immediately frozen at -80 °C. After thoroughly washing the intestinal contents under running water, the mucosal surface was macroscopically evaluated for pathological changes, and 1 × 1 cm sections were obtained and preserved in 10% neutral buffered formalin. A mucosal sample was also taken from the rectum using biopsy forceps (bREC), mimicking the diagnostic procedure performed in horses.

### Methods

#### Analysis of the SIgA content in the fecal and intestinal tract

The sample solutions were prepared for further analysis. After thawing at room temperature, fecal and intestinal content samples were prepared as described in the literature using our own modifications (Marr et al. 2020; Żak-Bochenek et al. 2022). From wet fecal samples, 1 g (± 0.05 g) of each sample was weighed after the sample had been thoroughly homogenized and suspended in 5 ml of phosphate-buffered saline (PBS) solution (pH ~ 7.4). Intestinal samples were diluted in PBS (1 g/5 mL). To thoroughly mix and release the proteins, the samples were subjected to the following procedure: shaking (3 min) and resting (15 min), twice. The samples were then centrifuged for 20 min at 1600 × g. The resulting centrifugation supernatant was collected, filtered through a syringe filter (syringe filter, 0.22 μm, ∅33 mm) and mixed with an inhibitor cocktail (Calbiochem Protease Inhibitor Cocktail Set I 539131, final dilution 1:100). The samples were centrifuged again for 15 min at 3260 × g. 600 µL of the supernatant was frozen at -80 °C for further SIgA analysis. The assay was performed using a commercially available ELISA kit (IgA Horse ELISA Kit; Abcam, Cat. No. ab190530) according to the manufacturer’s instructions. Samples of fecal supernatant and intestinal content were thawed at room temperature and then diluted 1:1500 (DUO, IL, CEC), 1:25 (COL), and 1:5 feces. The intra-assay CV reported in these studies was 1.69 ng/mL, and the inter-assay CV was 2.35 ng/mL.

#### Histopathological examination

Nine mucosal biopsy samples from each site (DUO, IL, CEC, COL, REC, and bREC) (54 in total) were fixed in 10% neutral buffered formalin for 48 h, processed for histopathology, and stained with hematoxylin and eosin (HE). Histopathological analysis was performed according to an established scheme based on the publication of Rötting et al. (2008) to analyze eosinophilic infiltration.

#### Immunohistochemistry of IgA + cells

Immunohistochemistry was performed on paraffin-embedded sections (5 μm thick) from mucosal biopsy samples from each site (DUO, IL, CEC, COL, REC, and bREC). Briefly, heat-induced antigen - retrieval was performed for 20 min at 97 °C in 10 mM sodium citrate buffer (pH 6.0; Dako Target Retrieval Solution) in a water bath. Endogenous peroxidase activity was quenched with a solution of 3% H2O2 for 10 min at room temperature. The sections were blocked in 2.5% normal horse serum (Vector ImmPRESS) for 20 min at room temperature and then incubated with goat anti-horse IgA antibody (Abcam, ab112868) for 60 min at room temperature (dilution 1:400). After washing with PBS, the samples were incubated with HRP-conjugated anti-goat IgG antibody (Vector ImmPRESS MP-7405) for 30 min at room temperature. The sections were washed again and incubated with chromogen (ImmPACT DAB Vector SK-4105) for 6 min in the dark. Lamina propria IgA + cells were counted using a Nikon Eclipse 80i microscope and video camera. Five areas were randomly chosen for each standardized area from the mucosal lamina muscularis line to the lumen of the intestinal tract (M1-M4 according to Rötting et al. 2008), and positively stained cells were counted. The results were expressed as positive cell numbers per 10,000 µm2.

#### Microbiome analysis

To extract DNA from microorganisms in luminal content samples, the QIAamp PowerFecal Pro DNA Kit (Qiagen) was used, following a standard protocol. Microbial DNA was isolated from mucosal tissue samples using the AllPrep DNA/RNA Mini Kit (cat no. 80204, Qiagen), and the DNA concentration was measured using a Quantus fluorometer (QuantiFluor^®^ dsDNA System, Quantum TM Fluorometer, Promega). DNA libraries for *16 S rRNA* sequencing were prepared using the QIAseq 16 S/ITS panel, targeting the variable regions V3-V4 and V7-V9 variable regions. For DNA derived from the luminal content sample set, a standard procedure was employed, whereas for DNA derived from the mucosal sample set, modifications were necessary. Specifically, 4 µL of DNA input was used instead of 1 µL and the first PCR cycle was extended from 12 to 20 cycles. To confirm the efficacy of DNA library preparation, a standard procedure was performed to assess the quality and quantity of DNA libraries. Library concentrations were measured using fluorometry (QuantiFluor^®^ dsDNA System, QuantusTM Fluorometer, Promega), and electrophoresis was conducted on a TapeStation with a High Sensitivity D1000 ScreenTape (Agilent Technologies). Not all samples yielded DNA libraries successfully (four REC/bREC-MS and six DUO/IL-LCS could not be obtained). However, for those libraries that were successfully prepared, library concentrations were normalized by diluting them to 2 nM (in UCP water, Qiagen), followed by paired-end sequencing on a MiSeq, 2 × 276, with the v3 MiSeq Reagent Kit (600 cycles), yielding FASTQ files.

Bioinformatics analysis was performed using Qiime2 with additional plugins, as previously described (Bolyen et al. 2019, Żak-Bochenek et al. 2022). To analyze microbiomes based on variable regions V3-V4 and V7-V9, Sidle employed a Qiime2 plugin that implements the SMURF algorithm (Fuks et al. 2018; Debelius et al. 2021). The SILVA 128 database is compatible with Sidle (Quast et al. 2013; Yilmaz et al. 2014).

#### Statistical analysis

Statistical analyses were performed using Python. For calculations and creating plots, the libraries ‘pandas’ (v1.2.5), ‘matplotlib’ (v3.4.3), ‘numpy’ (v1.21.2), ‘seaborn’ (v0.11.2) and ‘skbio’ (v0.5.6) (Hunter 2007; McKinney 2010; Harris et al. 2020; Reback et al. 2021; Waskom 2021; Rideout et al. 2024). Alpha diversity measures describe the diversity within a sample. Different alpha diversity metrics capture various aspects of diversity. The Shannon index considers both the number of different taxa and their evenness (Shannon 1948; MacArthur 1955). Low evenness occurs when one taxon dominates others, while high evenness is when all taxa are equally represented. Greater taxa diversity and higher evenness lead to a higher Shannon entropy value. Conversely, a low Shannon entropy value indicates either few different taxa or uneven distribution of taxa, indicating the dominance of certain taxa. The maximum value of Shannon entropy depends on the number of taxa and is achieved when all taxa are evenly distributed. Pielou’s evenness measures how equally individual taxa are represented in a sample (Pielou 1966). A high Pielou’s evenness suggests that all species are equally abundant, whereas a low index indicates significant disparities in species abundance. The index ranges from 0 to 1, where 0 means one taxa dominates entirely, and 1 means all taxa are represented equally. Faith’s Phylogenetic Diversity (Faith’s PD) is a measure of diversity that considers the phylogenetic relationships between taxa and is calculated as the sum of the branch lengths of the phylogenetic tree leading to the species present in a given ecosystem (Faith 1992). This means that two different taxa closely related contribute less to diversity than two species that are distantly related. A higher value thus indicates greater phylogenetic diversity in the sample In order to conduct a statistical comparison of alpha diversity measures (Shannon entropy, Pielou’s evenness, and Faith’s Phylogenetic Diversity) between intestine parts, as well as the concentration of SIgA in luminal content samples and IgA + cells in mucosal samples, and between two types of rectal sample acquisition (biopsy bREC vs. tissue REC-MS), the Kruskal-Wallis test from the ‘kruskal’ method was applied in the ‘scipy.stats’ library (v1.7.1) was applied (Virtanen et al. 2020). To evaluate differences between groups, if there were more than two, a post hoc Dunn test with Benjamini-Hochberg p-value correction from the ‘scikit_posthoc’ library ‘scikit_post hoc’ (v0.7.0) was used (Terpilowski 2019). Spearman’s correlation analysis was conducted to assess the relationship between alpha diversity measures and concentrations of SIgA and IgA + cells. Spearman’s correlation was used to compare alpha diversity measures between the two types of rectal samples collected from the same individual. These analyses utilized the ‘spearmanr’ function of the ‘scipy.stats’ library. Beta diversity captures the differences in the microbiome between samples. Weighted UniFrac is a measure of beta diversity that accounts for phylogenetic relationships along with the abundance of taxa (Lozupone et al. 2007). The result of beta diversity analysis is a distance matrix, which is then presented on a plot as the result of Principal Coordinate Analysis (PCoA). The Weighted UniFrac metric was employed to evaluate beta diversity, and distance matrices were calculated using the ‘q2-diversity’ function (Lozupone et al. 2007). To examine differences in distance matrices between groups, PERMANOVA with 999 permutations (bREC vs. REC-MS) from ‘skbio.stats.distance’ was applied. PERMANOVA with subsequent pairwise post hoc PERMANOVA tests was conducted alongside the Benjamini-Hochberg p-value correction for tests requiring multiple comparisons (DUO + IL vs. CEC vs. COL vs. REC). To assess the correlation between two distance matrices derived from the same intestinal part but different sample types (mucosal samples vs. luminal content samples), as well as from rectal biopsy and rectal mucosal samples, a Mantel test was performed using the ‘mantel’ function from the ‘skbio’ library. The test used the Spearman correlation method and considered a two-sided alternative hypothesis. A total of 9999 permutations were performed to calculate the p-value of the observed correlation coefficient. Differential abundance analysis refers to the assessment of differences in the number of various microorganisms in the microbiome. Sequencing data are inherently compositional, what means they measure relative abundances, allowing inferences about the proportions between different types of microorganisms but not about their absolute quantities (Morton et al. 2019). Due to this characteristic, appropriate statistical methods must be applied. To investigate which taxa differ significantly in abundance, differential analysis was performed based on a frequency table at the genus level using the ANCOM-BC2 (Analysis of Composition of Microbiomes with Bias Correction 2) (Lin and Peddada 2024). It is a statistical method that accounts for biases and compositional effects in microbiome data. The method incorporates bias correction techniques to control for false discoveries and improves statistical power by adjusting for covariates and allows for multi-group analyses (Lin and Peddada 2024). ANCOM-BC2 performs a log transformation of data with the addition of pseudo-counts to account for zero values, conducts sensitivity analysis on the impact of addition of pseudo-counts, and performs linear regression models on the bias-corrected log abundance table using the different pseudo-counts. As a result indicating those taxonomic groups that are likely to differ in abundance. The results were visualized on heat maps generated using Python software.

## Results

**Fig. 1:**
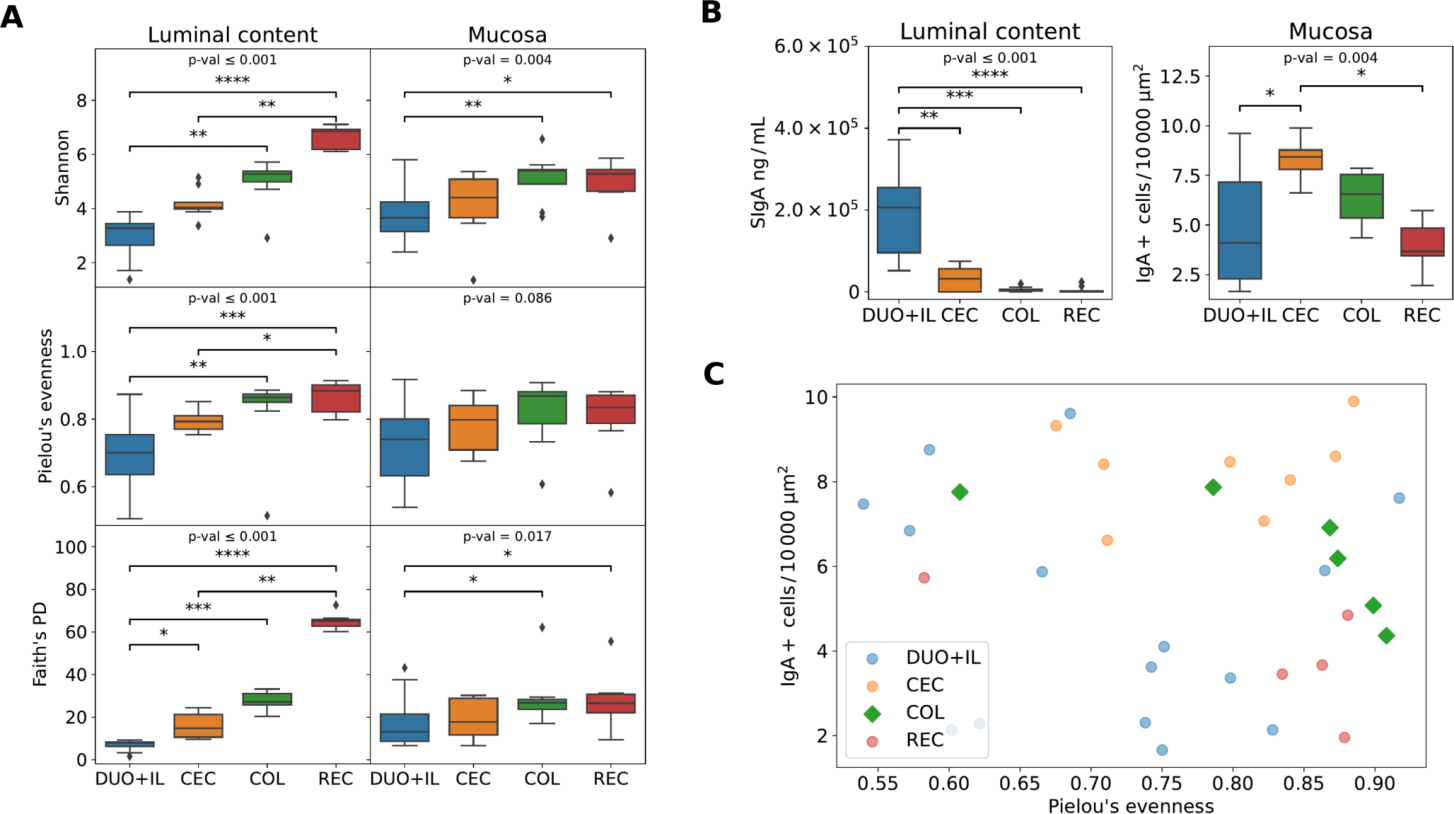
IgA and alpha diversity of the mucosal and luminal content microbiome. Legend: **A** Alpha diversity across different parts of the horse gut. The figure consists of six plots, presented as two panels (one for each sample type: luminal content and mucosa in columns) for each of the alpha diversity metrics (Shannon, Pielou’s evenness, and Faith’s Phylogenetic Diversity in rows) among four gut regions (DUO + IL, CEC, COL, REC). The Kruskal-Wallis test was used to test if there were statistically significant differences between groups, and *p*-values for these tests were provided on the plots. To identify which groups (DUO + IL, CEC, COL, REC) exhibited significant differences, a post hoc Dunn’s test with Benjamini-Hochberg *p*-value correction was performed (groups with statistically significant differences marked with symbols ✳ *p* ≤ 0.05, ✳✳ *p* ≤ 0.01, ✳✳✳ *p* ≤ 0.001, ✳✳✳✳ *p* ≤ 0.0001). **B** Two panels among parts of the horse gut (DUO + IL, CEC, COL, REC) in two types of samples, luminal content for SIgA (ng/ml) and mucosa for IgA + cells per 10 000 μm2. To identify which groups (DUO + IL, CEC, COL, REC) exhibited significant differences, a post hoc Dunn’s test with Benjamini-Hochberg *p*-value correction was performed (groups with statistically significant differences marked with symbols ✳ *p* ≤ 0.05, ✳✳ *p* ≤ 0.01, ✳✳✳ *p* ≤ 0.001, ✳✳✳✳ *p* ≤ 0.0001). **C** IgA vs. alpha diversity correlation: The only statistically significant correlation of Spearman’s correlation of alpha diversity in Pielou’s evenness with IgA + cells per 10 000 μm2 of mucosal samples of colon

**Fig. 2:**
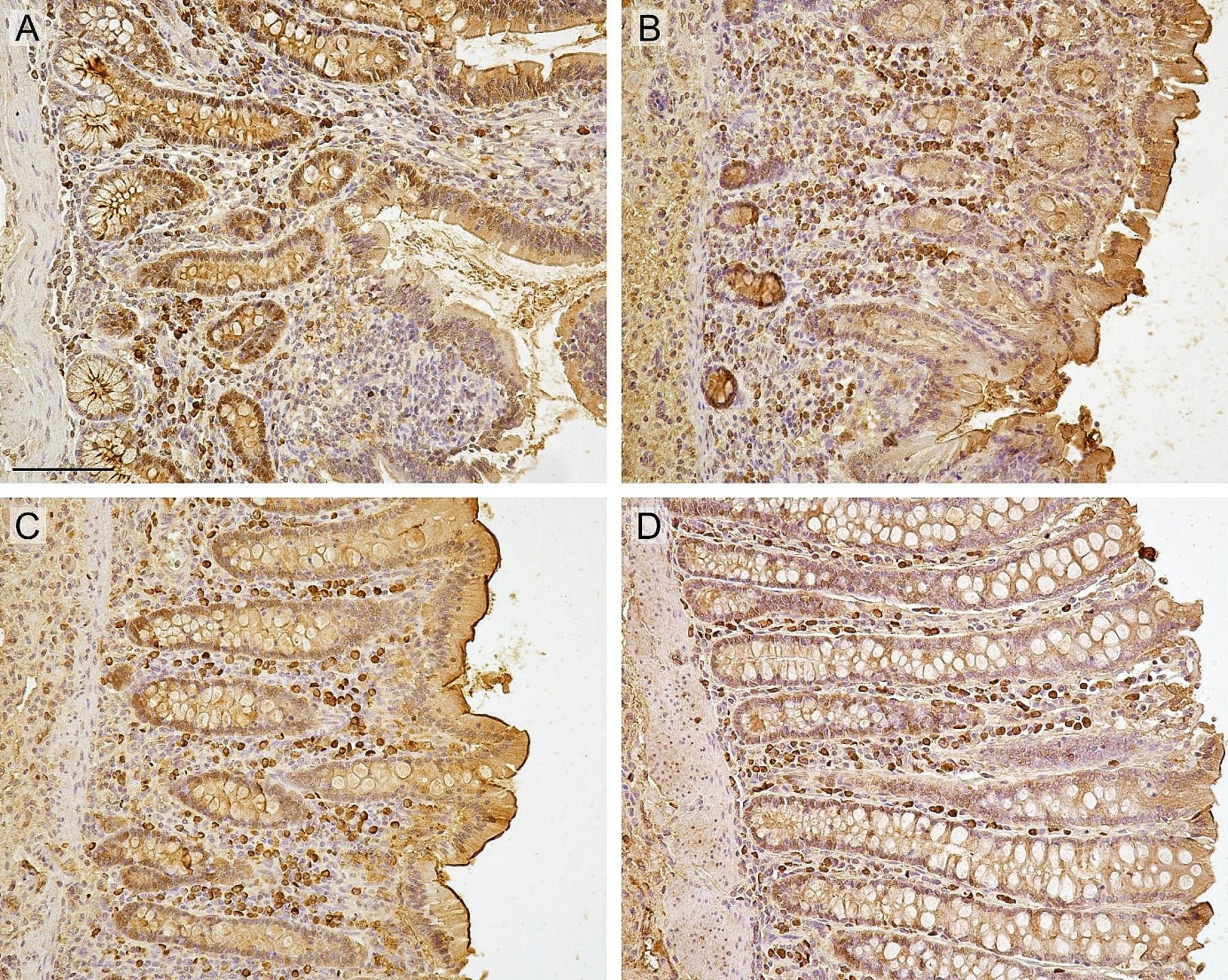
Immunohistochemical staining of IgA + cells. Legend: Immunohistochemical staining of IgA + plasma cells in the ileum (**A**), caecum (**B**), colon (**C**) and rectum (**D**) of the horse; scale bar 100 μm

Macroscopic evaluation of intestinal sections showed slight inflammatory changes, including mucosal congestion (horse numbers: 5 and 6), tapeworm infection (horse 1), and single ulcerations in the cecum (less than 0.8 cm in diameter) in two horses (2 and 9). Histopathological investigation showed no significant pathology in the samples examined and only a slight eosinophilic infiltration, which occurs in horses under physiological conditions. Analogous to the results presented by Rötting et al. (2008), the mean number of eosinophils per square millimeter increased according to the direction of the intestine: DUO > IL > CEC > COL, and cells predominated significantly in the basal part of the mucosa relative to the lumen part. Pathological cellular infiltration was not observed. Due to the smaller number of microbiome isolates from samples derived from the small intestine, the results of DUO and IL samples were combined for analysis and presented as “DUO + IL”. Post hoc pairwise PERMANOVA with 999 permutations showed no differences in beta diversity measured by Weighted UniFrac between DUO and IL samples, allowing them to be combined into one group (for LCS samples *p* = 0.436, for MS *p* = 0.306). The REC sample in the LCS group was conclusive with the fecal samples. A visible trend of increasing microbial diversity and evenness was observed during the transition from the small intestine to the rectum (Fig. 1A). This relationship was more pronounced in luminal content samples than in mucosal samples. The highest concentration of SIgA [ng/mL] was detected in DUO + IL-LCS The highest number of IgA + cells in the mucosa was observed in the CEC-MS samples, with decreased detection along the preceding and following segments of the intestine. The detailed data are presented in Figs. 1B and 2.

**Fig. 3:**
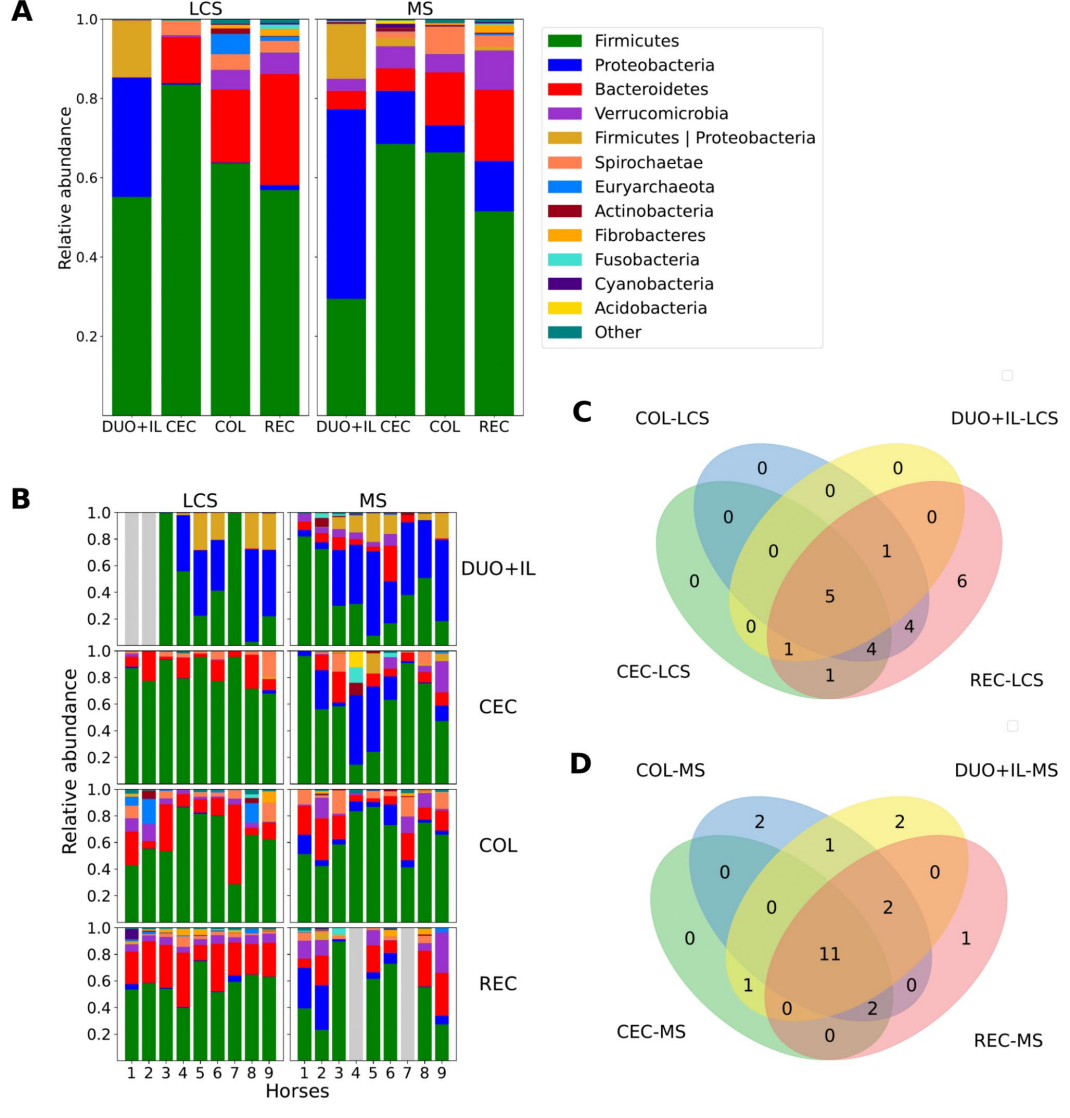
Relative abundance of Phyla across gut segments and sample types. Legend: **A** Averaged single taxa scores for individual fragments. **B** Results for a individual horse. Grey indicates a missing sample. Taxa whose relative abundance in any sample was less than 5% are filtered in ‘Other’. **C** Common taxa for each part of the gut at the cluster level, for LCS and **D** for MS

The only statistically significant correlation was Spearman’s correlation of alpha diversity in Pielou’s evenness with IgA + cells in the colon (COL) (*r*= -0.943, *p* = 0.0048). With increasing evenness, the number of IgA + cells decreased, but the results should be interpreted with caution because of the small sample size (Fig. 1C). The mean SIgA value in the fecal samples was 24.8 µg/ g of feces (SD = 39.2). In the LCS, the dominant phylum was *Firmicutes* (55% for DUO + IL, 83% for CEC, 64% for COL, and 57% for feces). The second most common phyla in DUO + IL samples were *Proteobacteria* (30%) and large intestine *Bacteroidetes* (12% in CEC, 18% in COL, and 28% in fecal samples). The next phylum for DUO + IL-LCS in terms of relative abundance was taxa showing ambiguity in classification: *Firmicutes* or *Proteobacteri*a (*Firmicutes| Proteobacteria*), which accounted for a significant proportion of relative abundance (up to 14%), and for the large intestine and fecal samples, *Verrucomicrobia*,* Spirochaete*,* Euryarchaeota*, and *Acidobacteria*, in varying orders across different parts of the intestine (results are shown in Fig. 3A and B). *Proteobacteria* predominated in MS samples for the small intestine (DUO + IL, 48%) and bREC (48%), while *Firmicutes* predominated in the other sections (69% in CEC, 66% in COL, and 52% in REC). In DUO + IL, *Firmicutes* was the second most dominant phylum. The remaining abundances are shown in Fig. 3A and B. Common taxa for each part of the intestine at the phylum level are shown in Fig. 3C and D.

**Fig. 4:**
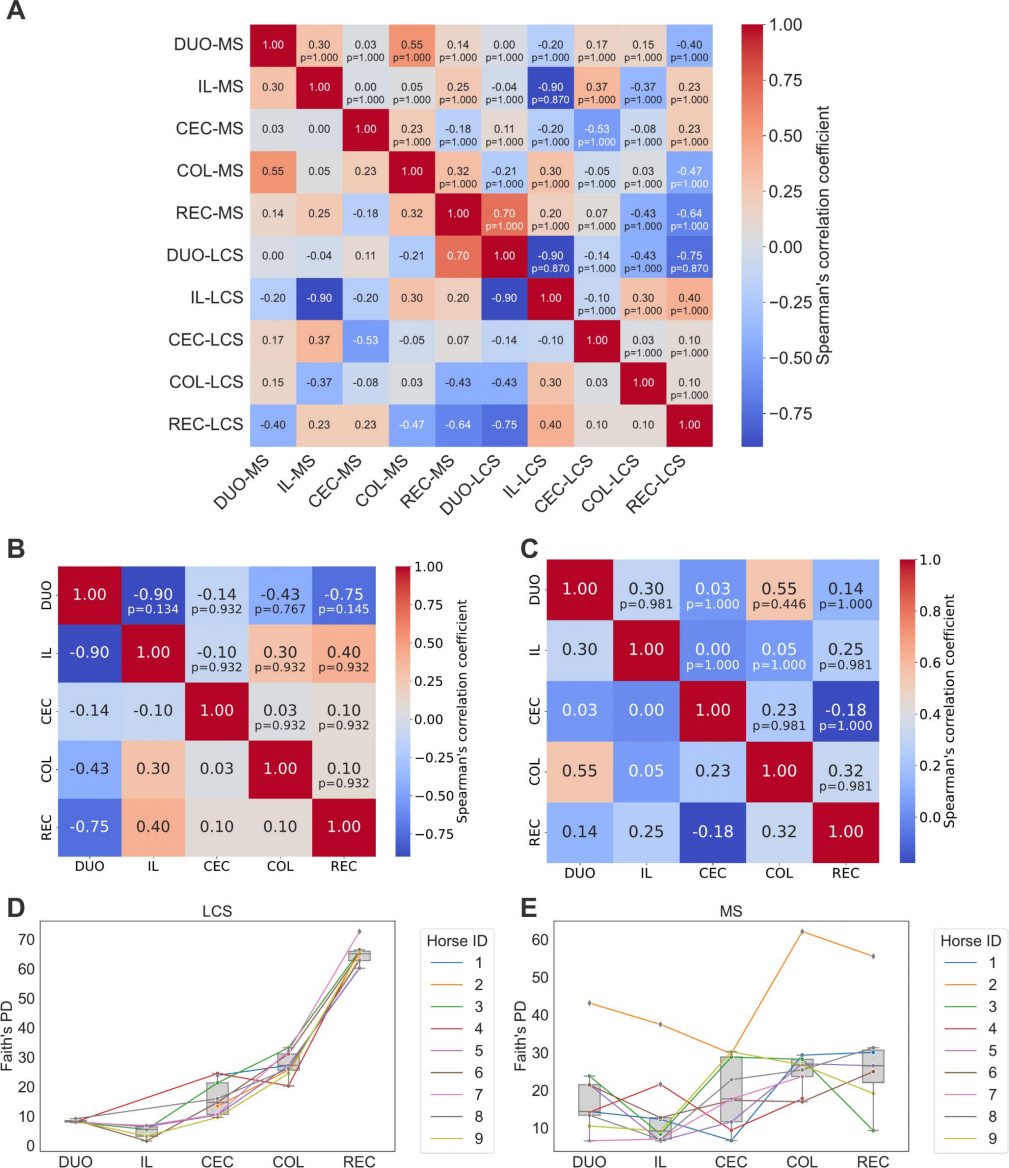
Correlation of the alpha diversity of the microbiome between gut segments and sample types. Legend: **A** Spearman’s correlation heatmap between intestinal segments (IL, DUO, CEC, COL, REC) originating from the same type of sample and between those segments originating from different types of sample (MS, mucosal sample versus LCS, luminal content sample). The central part of the cells displays the Spearman correlation coefficient value, and the bottom shows the corrected p-value (Benjamini-Hochberg FDR). No statistically significant correlations are observed between gut segments within the same sample type or between the same segment between two sample types. **B** The correlation of alpha diversity Faith’s PD between gut segments within LCS (as shown in the heat map in Panel A, lower right quadrant): There are no statistically significant correlations between the microbiome in luminal content between intestinal segments (DUO-MS vs. IL-MS vs. CEC-MS vs. COL-MS vs. REC-MS) in alpha diversity measured by Faith’s PD [p-value corrected (Benjamini-Hochberg FDR)]. **C** The correlation of alpha diversity Faith’s PD between intestinal segments within MS (as shown in the heatmap in panel A, the upper left quadrant): There are no statistically significant correlations between the microbiome in the mucosa between the intestinal segments (DUO-MS vs. IL-MS vs. CEC-MS vs. COL-MS vs. REC-MS) in alpha diversity measured by Faith’s PD. Line plots depicting changes in the Faith’s PD for each individual against the background of box plots. **D** A systematic increase in average alpha diversity can be observed, rising from IL-LCS towards REC-LCS. **E** The trend in changes in average alpha diversity is less systematic in samples derived from mucosa of different gut segments than in LCS

No statistically significant correlations were observed between the gut segments within the same sample type or between the same segment of the two sample types in alpha diversity measured using Faith’s PD (Fig. 4A, B, C). A systematic increase in the average alpha diversity was observed (Faith’s PD), rising from the DUO + IL-LCS to the REC-LCS (Fig. 4D). The trend in changes in average alpha diversity was less systematic in samples derived from the mucosa of different intestinal segments than in those derived from LCS (Fig. 4E).

**Fig. 5:**
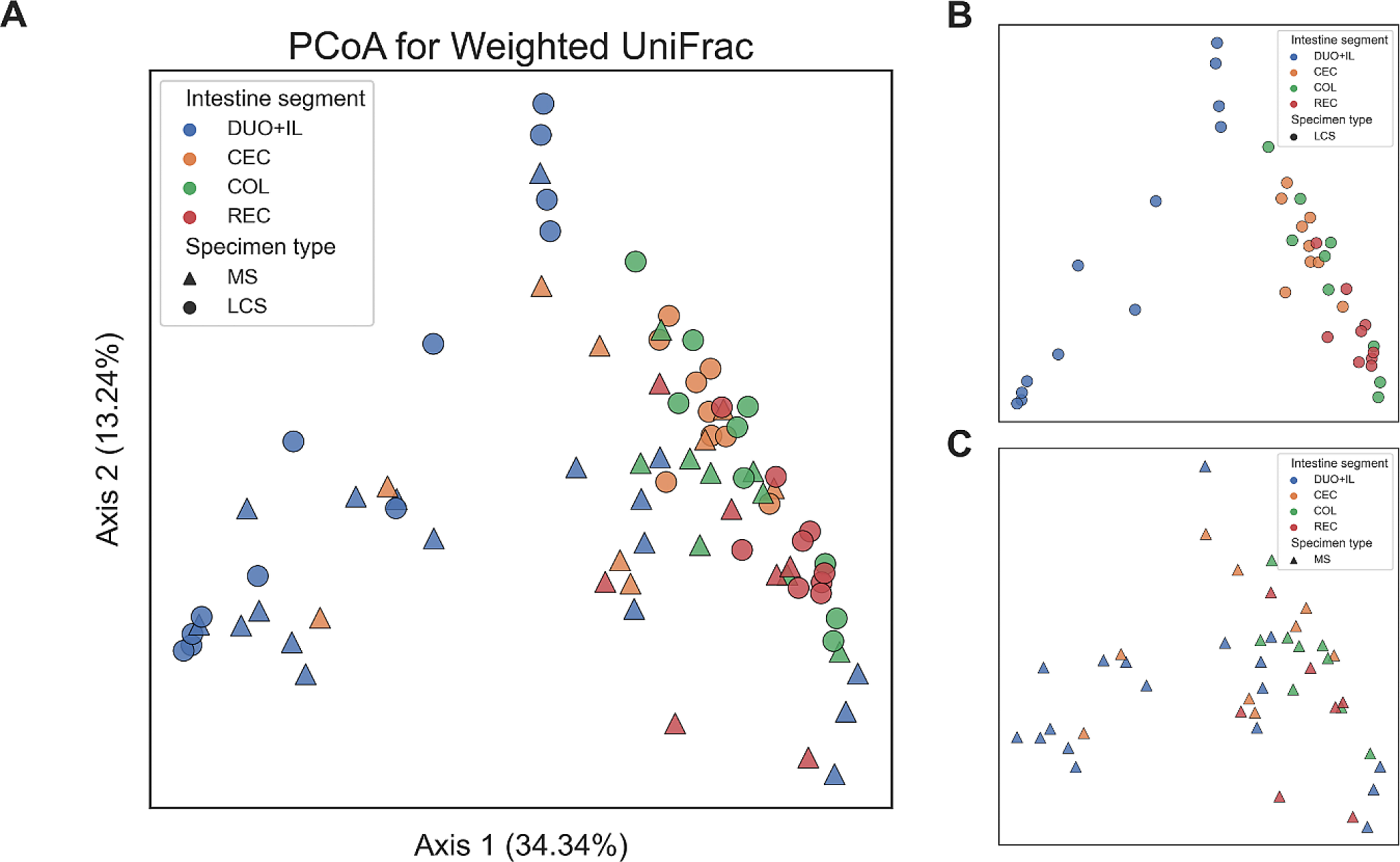
Principal Coordinate Analysis (PCoA) using Weighted UniFrac. Legend: **A** Depicting beta diversity between intestine segments (DUO + IL, CEC, COL, REC, blue, orange, green, and red, respectively) and between specimen types (LCS shown as circle and MS as triangle). For better visualisation of the distribution of samples from segments of the intestine within sample types, intestinal samples belonging to the LCS type were displayed separately in panel **B**, and samples belonging to the MS type were displayed separately in panel **C**

To assess which parts of the intestine differed in beta diversity using the Weighted UniFrac metric, a PERMANOVA test with 999 permutations was conducted. The test results for all groups in the LCS and MS samples indicated statistically significant differences, with p-values of 0.001 and 0.004, respectively (Fig. 5A, B,C).

To assess which parts of the intestine differed, post-hoc tests were conducted — pairwise PERMANOVA with 999 permutations, and p values were adjusted using the Benjamini-Hochberg method. It was shown that within the LCS, there were significant differences between pairs: CEC-LCS vs. COL-LCS (pcorr = 0.006), CEC-LCS vs. DUO + IL-LCS (pcorr = 0.0015), CEC-LS vs. REC-LCS (pcorr = 0.0015), COL-LCS vs. DUO + IL-LCS (pcorr = 0.0015), and DUO + IL-LCS vs. REC-LCS, but not COL-LCS vs. REC-LCS (pcorr = 0.084). Similarly, within the same segments of the intestine, but based on the type of the MS specimen type, it can be concluded that there are statistically significant differences only between the samples COL-MS vs. DUO + IL-MS (pcorr = 0.03), while between the remaining segments of the intestine, statistically significant differences were not observed (CEC-MS vs. COL-MS, pcorr = 0.122; CEC-MS vs. DUO + IL-MS, pcorr = 0.2376; CEC-MS vs. REC-MS, pcorr = 0.1335; COL-MS vs. REC-MS, pcorr = 0.522; DUO + IL-MS vs. REC-MS, pcorr = 0.057). To check whether distance matrices in beta diversity measured using Weighted UniFrac correlate within a given part of the intestine between the two types of samples (MS vs. LCS), a Mantel test was performed. There was a statistically significant correlation only within the COL part (Mantel test, rS COL = 0.504, *p* = 0.045, *n* = 9), whereas no significant correlation was detected between the distance matrices describing the beta diversity measured by Weighted UniFrac across other segments (Mantel test, rS DUO + IL = 0.18, *p* = 0.085, *n* = 12; rS CEC = 0.03, *p* = 0.868, *n* = 9; rS COL=-0.038, *p* = 0.88, *n* = 7).

**Fig. 6:**
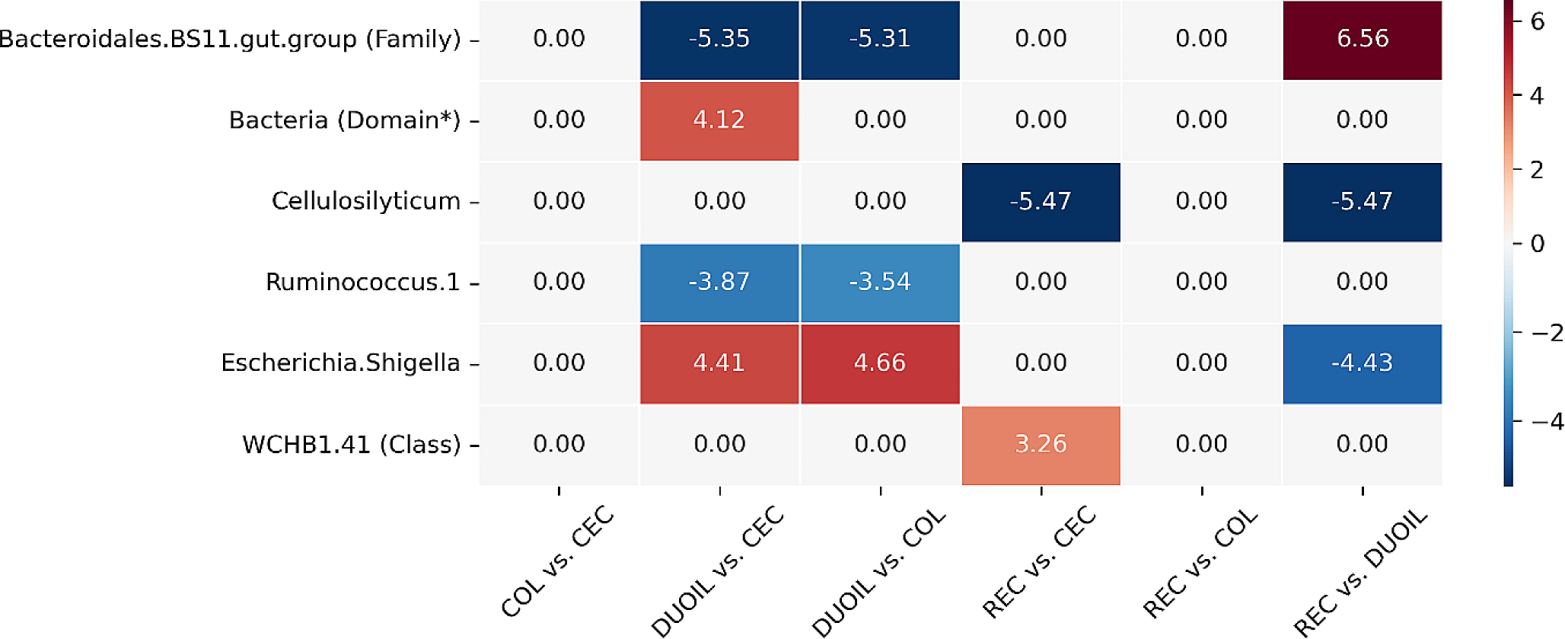
Differential abundance analysis in the parts of the intestinal tract in LCS in ANCOM-BC2. Legend: The heatmap shows differentially abundant taxa (p-value corrected < 0.05), insensitive to the addition of pseudo-counts, whose abundances in the LCS differ. The differential abundance analysis was conducted using ANCOM-BC2 based on the frequency table for genus level. For taxa where identification at the genus level was ambiguous, the lowest level of classification that was unambiguous is provided. The values are expressed as natural logarithms (lfc, log-fold change). Taxa for which no significant differences were found between the compared groups were normalised to a logarithmic difference equal to zero. For example: The logarithmic differences are as follows, for Bacteroidales.BS11.gut.group lfc =–5.35 DUO + IL vs. CEC, which means that e-5.35 equals approximately 0.00475, that is, the abundance of Bacteroidales.BS11.gut.group in the luminal content of the small intestine (DUO + IL) is about 210 times lower than in CEC

The results of the differential abundance (DA) analysis using ANCOM-BC2 revealed several statistically significant differences in the abundance of taxa in segments of the horse intestine sampled from the luminal content. DA was performed based on the frequency table at the genus level (Fig. 6).

In cases in which genus-level identification was not possible, the first available taxonomic level was indicated where classification was feasible. In the luminal content of the small intestine (DUOIL-LCS), the abundance of a taxa classified with confidence at the Family level — *Bacteroidales*.BS11.gut.group - is more than 210 times lower compared to its abundance in CEC-LCS, and more than 202 times less compared to COL-LCS, as well as more than 706 times less abundant in DUOIL-LCS than in REC-LCS. A significant difference in abundance was observed between DUOIL-LCS and CEC-LCS (over 61.5 times more abundant in DUOIL-LCS); however, the taxon marked on the figure as Bacteria (Domain*) has classification uncertainty at the phylum level (it cannot be distinguished whether the ASV sequence belongs to Phylum *Firmicutes* or *Proteobacteria*); therefore, this result should be treated as an artifact. In the luminal content of DUOIL and CEC, Genus *Cellulosilyticum* was more than 237 times more abundant than that in REC-LCS. In DUOIL-LCS, the genus Ruminococcus.1 was nearly 48 times less abundant than in CEC-LCS and almost 34.5 times less abundant than in COL-LCS. The *Escherichia.Shigella* genus in the luminal content of DUOIL-LCS is more than 82 times more abundant than in CEC, over 105 times more abundant than in COL, and almost 84 times more abundant than in REC. A taxon classified with confidence at the class level as WCHB1.41, belonging to the Phylum *Verrucomicrobia*, was over 26 times more abundant in REC-LCS than in CEC-LCS. No statistically significant differences in abundance were observed for the indicated taxa between the segments of the large intestine (CEC vs. COL and COL vs. REC). The results of DA analysis did not indicate significant differences in the abundance of any taxa between the intestinal parts sampled from the mucosa. In the bREC and REC-MS samples, only one significant change was observed in DA. The family *Spirochaetaceae* was 36 times less abundant in samples derived via biopsy from REC than in mucosal samples from REC.

**Fig. 7:**
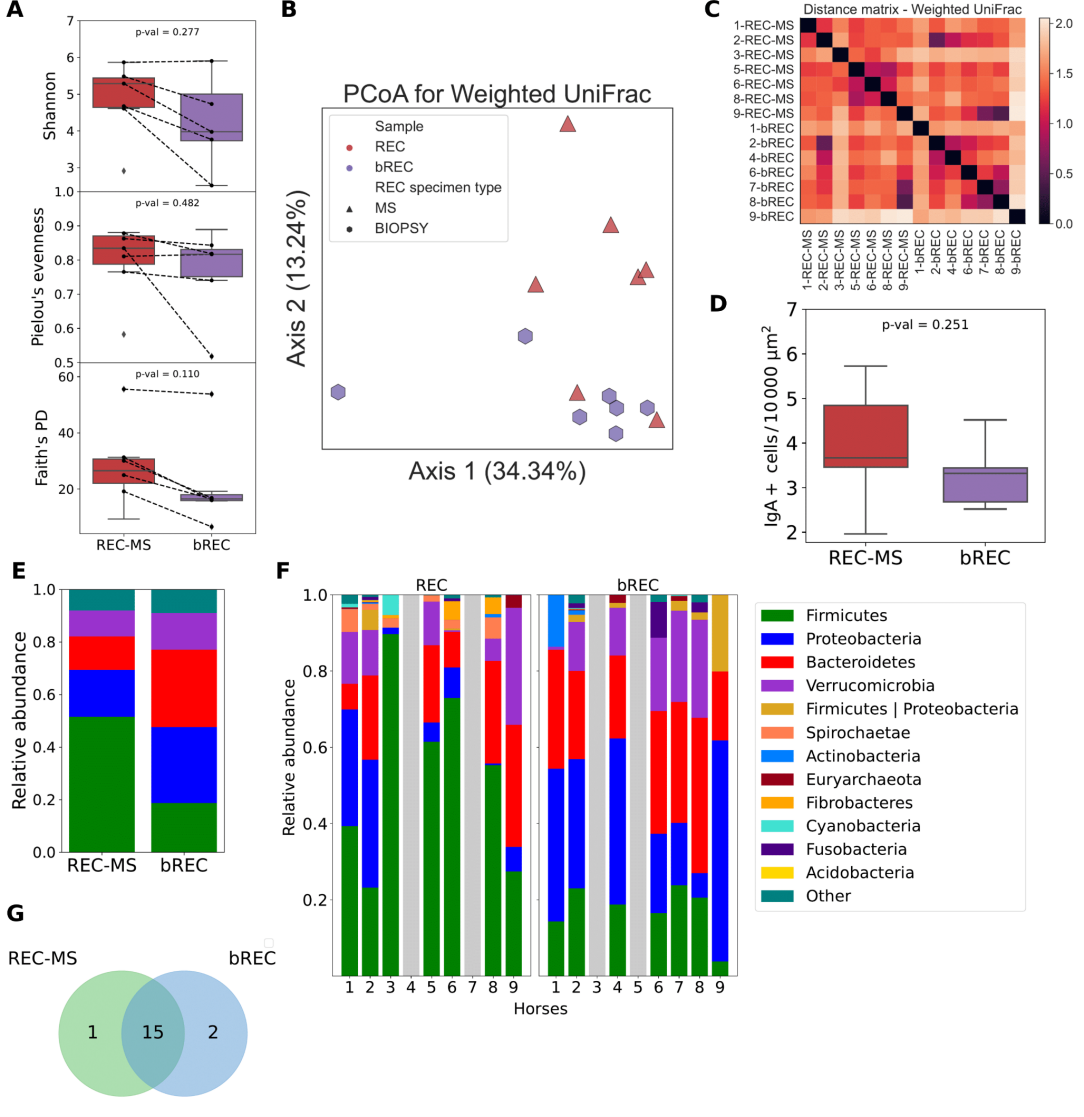
Comparison between REC-MS and bREC samples. Legend: **A** Alpha diversity for REC-MS (red) and bREC (purple). These types do not differ in alpha diversity, Shannon *p* = 0.277, Pielou’s evenness *p* = 0.482, and Faith’s PD *p* = 0.110, Kruskal-Wallis test. A strong Spearman correlation was observed between REC-MS and bREC in 5 sample pairs in the Shannon metric, rS = 0.999, *p* = 1.404 × 10–24; and strong, but statistically insignificant correlations were found in the Pielou’s evenness metric (rS = 0.6, *p* = 0.285) and in the Faith’s PD metric (rS = 0.7, *p* = 0.188). A cautious inference suggests that the alpha diversity of bREC samples might approximate that of REC-MS. However, this result should be interpreted cautiously due to the limited sample size. **B** Principal Coordinate Analysis (PCoA) using Weighted UniFrac, depicting beta diversity between REC-MS and bREC samples. No statistically significant differences were found, *p* = 0.059, PERMANOVA test of 999 permutations; however, this result is at the threshold of statistical significance. **C** For horses 1, 2, 6, 8, and 9, in the sample pairs (REC-MS and bREC), the distances between distance matrices for Weighted UniFrac are not the smallest, suggesting that they may not closely approximate each other’s beta diversity. Additionally, there is no significant correlation between distance matrices in these pairs (Mantel test results: rS = 0.539, *p* = 0.136). **D** REC-MS and bREC do not differ in IgA + cell concentration per 10,000 µm2, *p* = 0.251, Kruskal-Wallis test, limitation is small sample sizes, REC-MS *n* = 5, bREC *n* = 5. **E** Average relative abundances at the phylum level in groups REC-MS and bREC. **F** Phyla relative abundances for individual specimens, for REC-MS and bREC. The grey bars indicate the absence of a sample. **G** At the phylum level, 15 taxa were identified as common to the samples REC-MS and bREC. One taxa are unique for REC-MS, and two are unique for bREC

The two types of REC segment samples (REC-MS and bREC) did not differ significantly from each other in any of the presented alpha diversity metrics (Fig. 7A-G).

The Kruskal-Wallis test was applied, Shannon’s *p* = 0.277, Pielou’s evenness *p* = 0.482, and Faith’s PD *p* = 0.110. Additionally, for samples that had their REC-MS and bREC pairs (five pairs of samples: horses 1, 2, 6, 8, and 9), Spearman’s correlation coefficients were calculated. A strong correlation was found between REC-MS and bREC samples in the Shannon metric (rS = 0.999, *p* = 1.404 × 10–24; and strong, but statistically insignificant correlations were found in Pielou’s evenness metric (rS = 0.6, *p* = 0.285) and Faith’s PD metric (rS = 0.7, *p* = 0.188). It can be cautiously inferred that the alpha diversity of the bREC samples may approximate that of REC-MS. These results should be interpreted with caution because of the small sample size. No statistically significant differences in beta diversity were found between the types of samples, with a PERMANOVA test of 999 permutations, *p* = 0.059; however, this result was not statistically significant. The distances in the distance matrix for Weighted UniFrac were also examined. It is not the case that in sample pairs (REC-MS and bREC, for horses 1, 2, 6, 8, and 9) the distances are the smallest, which may indicate that they are not a good approximation of beta diversity. A Mantel test was performed to assess whether the distance matrices in the Weighted UniFrac of bREC samples correlated with the distance matrix of REC-MS and to investigate whether bREC samples may approximate the beta diversity of REC-MS samples. The comparison was performed on five pairs of samples (horses 1, 2, 6, 8, and 9). There was a moderate positive correlation between the two groups. The value of the Spearman correlation coefficient, rS = 0.539, suggests some structural similarity between the groups. However, this result does not indicate a statistically significant difference between the two types of samples (five pairs of bREC-REC-MS samples), as *p* = 0.136. This result suggests that there is insufficient evidence to confirm that both groups are approximations of each other. However, a larger sample size may be necessary to obtain a more conclusive assessment. The REC-MS samples and bREC did not differ significantly in terms of the concentration of IgA + cells (Kruskal-Wallis test, *p* = 0.251). At the phylum level, 15 taxa were identified as being common to the REC-MS and bREC samples. One taxon is unique to REC-MS and two are unique to bREC.

## Discussion

SIgA acts as the first line of defense against pathogens, facilitates colonization of the mucus surface by the commensal microbiota, and regulates immune homeostasis. The presence of SIgA in the mucosal “killing zone” is important not only for the inhibition of pathogens but also for their interaction with the microbiome (Chase and Kaushik 2019). The first is performed via neutralization and immobilization functions. In addition to pathogen neutralization, SIgA regulates the composition of the gut microbiota and plays a role in maintaining the homeostatic relationship between the intestinal microbiota and the host (Pietrzak et al. 2020). According to Yang et al. (2020) the abundance of fecal IgA depends on the colonization of the intestine by the microbiota. SIgA plays a critical role in controlling the composition of the gut microbial community and maintaining a diverse and stable gut microbiota. However, this is not a one-sided relationship; many investigations have indicated that particular strains of the microbiome can induce IgA production (Pabst and Slack 2020). Furthermore, in germ-free mice, IgA production was barely detectable in the small intestine but was not detected in the large intestine (Yanagibashi et al. 2013). The investigations by Yang et al. (2020) highlight the critical importance of microbial strains in driving phenotypic variation in the mucosal immune system and provide a strategy to modify an intestinal immune phenotype, including robust IgA production. The most commonly described relationship is related to the influence of the microbiota on IgA production in the small intestine of mice by inducing the development of intestinal-associated lymphoid tissue, such as Payer’s patches (Suzuki and Fagarasan 2008). In horses, Payer’s patches can be distributed throughout the gastrointestinal tract, including the cecum and colon, unlike in other animal species, and this interaction can also occur outside the small intestine (Liebler-Tenorio and Pabst 2006). Our analysis showed no significant correlation between SIgA levels in the luminal content or the number of IgA + cells in the sections and the key elements of the horse’s gut microbiome. However, within a large colon, with increasing Pielou’s evenness, the number of IgA + cells decreases, but the result should be interpreted with caution due to the small sample size. Immunohistochemical examination using anti-equine IgA antibodies allowed imaging of the lamina propria of plasmocytes responsible for SIgA production (Fig. 2). Furthermore, positive results were observed in epithelial cells and luminal contents, confirming data from the literature on the role of epithelial cells in IgA transport and their presence in their contents (Chase and Kaushik 2019). The high level of SIgA in the LCS in relation to the low number of small intestinal IgA + cells in the study presented here may be due to the extraintestinal origin of SIgA, released into the duodenum in the form of bile (Pabst and Slack 2020).

Equine digestive physiology is characterized by rapid gastric transit, rapid but intense enzymatic digestion in the small intestine, and long and intense microbial fermentation in the large intestine (Santos et al. 2011). In recent years, many scientific papers have investigated both the fecal microbiome (physiological and in different states of disease or with different maintenance) and the microbiome in particular sections of the equine gastrointestinal tract (Costa et al. 2012, 2015; Ericsson et al. 2016; Su et al. 2020; Reed et al. 2021; Chaucheyras-Durand et al. 2022). In herbivores, maintaining a proper gastrointestinal microbiota is essential to ensure proper digestion of the fiber, which is the basis of their diet, and to be able to use short-chain fatty acids as a source of energy. Previous studies, based on the diversity, richness, and composition of the microbiome, have indicated the possibility of distinguishing between two different ecosystems of the equine gastrointestinal tract, the small intestine and the large intestine (Costa et al. 2015; Ericsson et al. 2016; Su et al. 2020). However, in the present study, the results of beta diversity analysis revealed the presence of a third distinct ecosystem, the cecum, which was also noted by Reed et al. (2021). The study presented here was able to confirm the thesis, as it showed significant differences in the amount of SIgA in the cecum content in relation to the other fragments.

An important question asked at the study design stage was to what extent a stool sample could reflect the condition of the gastrointestinal tract as a non-invasive method. The study presented here confirmed the literature data indicating the similarity of the microbiome between large colon and fecal samples. (Costa et al. 2012, 2015; Ericsson et al. 2016; Su et al. 2020; Reed et al. 2021; Chaucheyras-Durand et al. 2022). In addition, the study was the first to allow confirmation of an analogous relationship for SIgA. For both the microbiome and SIgA, however, it is unclear to what extent significant pathologies in the small intestine can be reflected in the faeces. However, this question requires further research.

To assess the differences in composition between the microbial communities in the LCS and MS, PCoA was performed using all samples.For both sample types, a similar distribution and differences between the small and large intestines were observed for analogous regions, confirming the results of Ericsson et al. (2016). In addition, in the context of alpha diversity, the MS samples showed a trend similar to that of the LCS samples; however, the trend was not statistically significant. As in the Ericsson et al. (2016) and Arroyo et al. (2020) studies, there were no significant correlations between the results obtained for the different sample types (MS vs. LCS) in each region.

Comparing the relative frequency of significant phylum in luminal content samples and mucosal sections, the authors noted that the *Proteobacteria* phylum dominated in MS in small intestine and ceacum samples, but not it LCS samples. It may suggest that representatives of this phylum may be located at these locations, not in the dense part of the mucus but closer to the intestinal epithelial cells. The relative increase in *Proteobacteria* tended to come at the expense of the phylum *Firmicutes*, which, by analogy, could be located in the mucus layer, away from the mucosa, and could be flushed away at the stage of rinsing the MS samples with sterile saline. An increase in the abundance of *Proteobacteria* in feces is considered a marker of dysbiosis in humans; therefore, in light of the above findings, it seems interesting to determine whether it is actually the phylum that begins to dominate the gastrointestinal tract, or whether there are structural changes within the mucosa and the barrier leading to their unsealing and release of bacteria. An increase in fecal *Proteobacteria* occurs in humans with inflammatory and cancerous diseases, which could be related to the removal of the mucosal barrier (Shin et al. 2015). Arroyo et al. showed that colitis in horses results in a decrease in the abundance of species belonging to the phylum *Firmicutes* and a predominance of *Proteobacteria* and pathogenic bacteria (Arroyo et al. 2020). Based on this research, one hypothesis is that *Firmicutes* may be mechanically flushed during the dilution of digestive contents and increase peristalsis during the course of diarrhoea (Arroyo et al. 2020). The potential contact of *Proteobacteria* with the lamina propria may be evidenced by the fact that this phylum is often described in the context of bacteria coated with specific SIgA, and their production requires close contact between cells and bacteria (Mirpuri et al. 2014). However, this appears to have a positive effect on the defense functions of the gastrointestinal mucosa but requires further research.

An important limitation of the study was the number of animals studied, although the group was larger than in other studies. More studies are needed to determine whether small intestinal pathologies are reflected in feces and how this is the case for colitis (inflammatory bowel disease). Further research by the authors will continue to investigate gastrointestinal pathologies in correlation with the microbiome and immune status in horses.

## Conclusion

In horses, as an example of non-ruminant herbivores, two different ecosystems can be distinguished in the gastrointestinal tract, that is, the small intestine and large intestine. The small intestine in relation to the large colon is characterised by fewer IgA + cells, more SIgA in the intestinal contents and a less diverse microbiome. However, the cecum appears to be the third separate ecosystem, with high number of IgA + cells and diverse microbiome. The fecal sample available for noninvasive collection reflects the current state of the large colon, both in terms of microbiome and SIgA content; however, the extent to which it may be affected by dysbiosis of the small intestine. Appropriately collected biopsies (mucosal samples) can also be used in the diagnosis of intestinal microbiota but require careful interpretation and more extensive studies to establish reference standards. Further studies are needed to assess the usefulness of fecal SIgA assessment in the pathological conditions of the small and large intestines of horses.

## Data Availability

The datasets supporting the conclusions of this article are available in the BioProject repository (https://www.ncbi.nlm.nih.gov/bioproject/1096964).
